# Injuries caused by fish to fishermen in the Vale do Alto Juruá, Western Brazilian Amazon

**DOI:** 10.1590/0037-8682-0495-2018

**Published:** 2019-12-20

**Authors:** Tamires Nascimento da Costa, Tiago Ricardo Fernandes Jacó, André Luis da Silva Casas, Paulo Sérgio Bernarde

**Affiliations:** 1 Universidade Federal do Acre, Programa de Pós-Graduação Stricto Sensu em Ciências da Saúde na Amazônia Ocidental, Rio Branco, AC, Brasil.; 2 Universidade Federal do Acre, Campus Floresta, Núcleo de Ictiologia do Vale do Alto Juruá, Cruzeiro do Sul, AC, Brasil.; 3 Universidade Federal do Acre, Campus Floresta, Laboratório de Herpetologia, Cruzeiro do Sul, AC, Brasil.

**Keywords:** Epidemiology, Venomous fish, Injuries, Envenoming, Fishermen, Occupational hazard

## Abstract

**INTRODUCTION:**

This study aimed to document injuries caused by fish among professional fishermen in the Western Brazilian Amazon.

**METHODS:**

We undertook a descriptive, retrospective study, involving 51 professional fishermen, to determine clinical, epidemiological, and therapeutic aspects of their injuries.

**RESULTS:**

Among 51 fishermen interviewed, most injuries were due to *mandi* (*Pimelodus* spp.), and the hands were the most injured region, resulting in pain and bleeding in all cases.

**CONCLUSIONS:**

Our study findings confirm the morbidity of fish-related injuries, and reaffirm the need for relevant information regarding prevention and injury management.

Injuries to humans caused by freshwater or marine fish have been reported to be very common^1^. Active injuries are characterized as venomous when fish such as freshwater stingrays and catfish^1^use their stinger or spines to inoculate toxins, causing lacerations to the integument and envenomation. Traumatic injuries comprise fish bites from *piranhas* and *traíras*, or electric fish that discharge electric shocks^1^. Other indirect injuries can occur when a victim ingests venomous fish such as pufferfish that have poison in their gut and gonads, or through the consumption of fish contaminated with bacteria, toxic plants, or hazardous chemical substances such as mercury^1^.

Injuries generated by aquatic animals are common in Brazil, although underreported^1^ ^,^ ^2^. Injured patients usually seek health services only when the clinical condition worsens. In the Northern region of Brazil, many injuries to humans from aquatic animals occur in distant places that are isolated from urban centers, leaving victims with limited possibilities of medical care^1^ ^,^ ^2^ ^,^ ^3^.

Health professionals residing in fishing communities find injuries from aquatic animals a common occurrence in their professional lives (e.g., in removing hooks from fish or fish from nets, and in handling or transporting fish)^4^ ^-^ ^6^. A lack of information concerning appropriate treatment may encourage injured fishermen to use alternative treatment for symptom relief from their injuries, such as medicinal plants^4^ ^-^ ^5^.

Considering the importance and severity of these injuries, we identified and described injuries caused by fish among professional fishermen in the municipality of Cruzeiro do Sul (Acre State, Brazil). We also describe the clinical profile, and the epidemiological and therapeutic aspects in this population.

In this descriptive and retrospective study, we used a questionnaire to evaluate the injuries caused by fish in 51 professional fishermen in Cruzeiro do Sul city. The Juruá river is a source of income for the riverside population and for fishermen through fishing activities. Injuries while fishing have been defined as envenoming or mechanical trauma caused by fish through bites, stingers, and spines, and included electric discharges.

Data collection occurred between September and December 2017, through interviews with fishermen from the Z-1 fishermen's community and the Resende de Souza Lima fish market. This study was approved by the Ethics Research Committee of the Northern Educational Union (CEP/UNINORTE n^o^. 2,092,520).

The questionnaire included general questions concerning fish-related injuries and the nature of the first aid performed at the time of the injury as well as questions concerning: (1) qualitative variables: sex, level of education, activity being undertaken at the time of injury, the time of day the injury was sustained (morning, afternoon, or evening), seasonality (rainy or dry), body region affected, the species of fish involved in the injury (for identification purposes, printed images of the main fish species that are present in the region were shown), symptoms, sequelae, medical care, alternative treatments, and injury site, and; (2) quantitative variables: age, how long the fisherman had practiced fishing activity and the number of times a fish-related injury had occurred for each individual. The fish species involved in the injuries were identified at the lowest possible taxonomic level by an ichthyologist taxonomist, based on fish specimens collected in the Upper Juruá region and deposited in the Laboratory of Anatomy and Comparative Physiology of the UFAC Campus Floresta (Cruzeiro do Sul) collection.

In total, 51 professional fishermen were interviewed, reporting 204 injuries, with all interviewees being males aged between 20 and 77 years (average, 47.3 years), and their years spent fishing professionally ranged from 6-45 years (average, 27.2 years). The data showed a low level of schooling, with 70.6% of interviewees having had an incomplete elementary education, 9.8% having completed secondary education, and 19.6% being illiterate. 

Most venomous injuries (52.9%) were due to *mandi* (*Pimelodus* spp.), 9.8% were due to stingrays, and 10.3% other pimelodid fishes to a lesser extent (Table 1 and Figure 1). The species most involved with causing injury were *piranha* (*Pygocentrus nattereri* and *Serrasalmus* spp.) (15.2%), *traíra* (*Hoplias* cf. *malabaricus*) (3.4%), *gata* (Cynodontidae) (2%), *piau* (Anostomidae) (1.5%), *matrinxã* (*Brycon* sp.) (1%), *poraquê* (*Electrophorus electricus*) (1%), *bacu* (*Lithodoras dorsalis*) (1%), and *candiru* (*Vandellia* sp.) (0.5%) (Table 1 and Figure 1). Electric discharges caused by *poraquê* occurred when fishermen were in the water. *Candiru* bites also occurred when fishermen were in the water, and bites occurred when handling *matrinxã*.

**TABLE 1:** Frequency of fish-related injuries, according to interviews with fishermen in the Vale of Alto Juruá.

Order / Family / Species	Popular name	Frequency
MYLIOBATIFORMES: POTAMOTRYGONIDAE		
*Paratrygon aiereba* (Müller & Henle, 1841)		
*Plesiotrygon iwamae* (Rosa, Castello & Thorson, 1987)		
*Potamotrygon motoro* (Müller & Henle, 1841)	Freshwater stingray	20 (9.8%)
*Potamotrygon orbignyi* (Castelnau, 1855)		
*Potamotrygon scobina* (Garman, 1913)		
CHARACIFORMES: SERRASALMIDAE		
*Pygocentrus nattereri (*Kner, 1858)		
*Serrasalmus rhombeus* (Linnaeus, 1766)		
*Serrasalmus elongatus* (Kner, 1858)	*Piranha*	31 (15.1%)
*Serrasalmus eigenmanni* (Norman, 1929)		
*Serraslmus spilopleura* (Kner, 1858)		
*Piaractus brachypomus* (Cuvier, 1818)	*Pirapitinga*	1 (0.5%)
CHARACIFORMES: ERYTHRINIDAE		
*Hoplias* cf. *malabaricus* (Bloch, 1794)	*Traíra*	7 (3.4%)
CHARACIFORMES: CYNODONTIDAE		
*Cynodon gibbus (*Spix & Agassiz, 1829)		
*Hydrolycus armatus* (Schomburgk, 1841)		
*Hydrolycus scomberoides* (Cuvier, 1816)	*Gata / Cachorrão*	3 (1.5%)
*Rhaphiodon vulpinus* (Spix & Agassiz, 1829)		
*Roestes molossus* (Kner, 1858)		
CHARACIFORMES: ANOSTOMIDAE		
*Leporinus trifasciatus* (Steindachner, 1876)		
*Leporinus fasciatus* (Bloch, 1794)	*Piau*	3 (1.5%)
*Laemolyta taeniata* (Kner, 1859)		
*Schizodon fasciatus* (Spix & Agassiz, 1829)		
CHARACIFORMES: CHARACIDAE		
*Brycon* sp.	*Matrinxã*	2 (1%)
SILURIFORMES: PIMELODIDAE		
*Brachyplatystoma flavicans* (Castenau, 1855)	*Dourada*	2 (1%)
*Hemisorubim platyrhynchus* (Valenciennes, 1840)	*Pimpão*	1 (0.5%)
*Sorubim lima* (Bloch & Schneider, 1801)	*Bico-de-Pato*	10 (4.9%)
*Pseudoplatystoma punctifer* (Castelnau, 1855)	Surubim	
*Pseudoplatystoma fasciatum* (Linnaeus, 1766)		7 (3.4%)
*Pimelodus blochii* (Valenciennes, 1840)	*Mandi*	108 (52.9%)
*Pimelodus* sp.		
SILURIFORMES: AUCHENIPTERIDAE		
*Auchenipterus ambyacus* (Fowler, 1915)	*Lustrosa*	4 (2%)
SILURIFORMES: DORADIDAE		
*Lithodoras dorsalis* (Valenciennes, 1840)	*Bacu*	2 (1%)
SILURIFORMES: TRICHOMYCTERIDAE		
*Vandellia* sp.	*Candiru*	1 (0.5%)
GYMNOTIFORMES: GYMNOTIDAE		
*Electrophorus electricus* (Linnaeus, 1766)	*Poraquê*	2 (1%)
**Total**		**204 (100%)**

**FIGURE 1:**
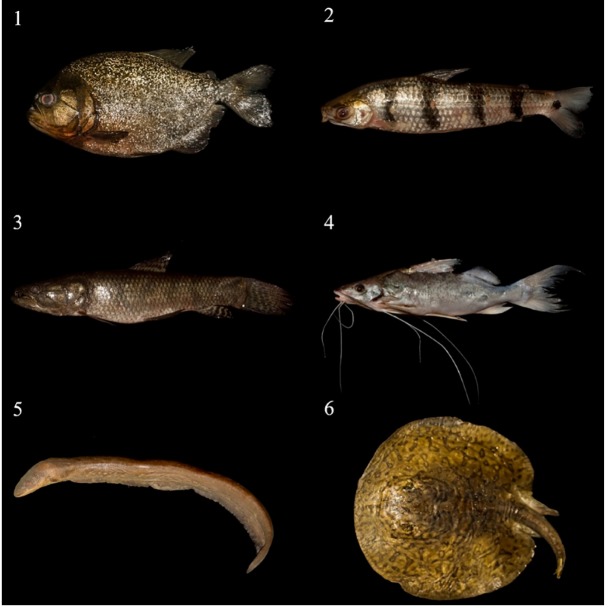
Examples of some species of fish that caused injuries among the fishermen. 1. *Piranha* (*Pygocentrus nattereri*) (length of specimen photographed, 265 mm); 2. *Piau* (*Schizodon fasciatus*) (length, 255 mm); 3. *Traíra* (*Hoplias* cf. *malabaricus*) (length, 333 mm); 4) *Mandi* (*Pimelodus blochii*) (length, 168 mm); 5. *Poraquê* (*Electrophorus electricus*) (length, 940 mm), and; 6 *Arraia* (*Potamotrygon* sp.) (length, 385 mm). Images by Luis Felipe Magalhães de Carvalho.

The fishermen identified 17 types of fish that had caused them injuries. These fish were classified according to their popular names that were not necessarily representative of their biological species. In total, 33 species of fish were identified from their classification (Table 1) with potential to cause injury.

Most interviewees indicated that injuries occurred when handling fish while removing fishing gear (97%), practicing spearfishing (2%), or during leisure fishing activities (1%). In terms of the time of day, 46.6%, 41.7%, and 11.7% of the injuries were sustained in the afternoon, morning, and evening, respectively. 

The upper limbs were the most affected sites of injury, with 60.3% of injuries sustained to the hand, caused by almost all of the identified fish species, except the *candiru*. *mandis*, *piranhas*, *bicos de pato*, *lustrosas,* and *douradas* were responsible for 3.4% of the injuries to the arms. The lower limbs were also injured, and 33.3% of injuries located on the feet were associated with stingrays, *mandis*, *piranhas,* and *poraquês*; 1% of injuries were located on the thigh, caused by *mandis*, 1% of *piranhas-* and *lustrosas-*associated injuries affected the legs, 0.5% of *mandi*-related injuries were located in the gluteal region, and 0.5% of injuries located in the abdomen were due to *candirus*. The interviewees reported signs and symptoms such as pain and hemorrhage (100%), edema and erythema (22.1%), fever (8.8%), necrosis and ulceration (7.8%), and syncope (0.5%).

Of the injured fishermen, 84% did not seek medical attention, whereas 16% sought medical treatment for injuries sustained due to freshwater stingrays, *mandis,* and *piranhas*, and were treated at the Juruá Regional Hospital in Cruzeiro do Sul. 

However, 22.5% of injured fishermen used alternative treatment to relieve signs and symptoms, as well as to treat complications (Table 2). Of these, 11.8% involved the use of termite mound smoke to treat the injured region, 2% involved condensed milk, 1.5% involved *açacu* milk, with both milk types used for wounds related to freshwater stingray injuries (1.5%). Furthermore, 1% of injured fishermen used mercurochrome to treat two injuries due to *mandis*, and 0.5% used mercurochrome to treat injuries caused by other fish species.

**TABLE 2:** Alternative remedies according to the popular name of the relevant fish.

Fish	Alternative remedies	Frequency	%
*Mandi*	Place the mandi’s eyes on the wound	1	0.5%
Stingray	Coffee grounds with cotton tea	1	0.5%
Stingray	Açacu (*Hura crepitans*) milk	3	1.5%
Stingray	Watermelon root	1	0.5%
Stingray	Coffee powder	1	0.5%
Stingray	*Leite condensado* (condensed milk)	4	2.0%
Stingray	Place the wound in contact with a human vagina	1	0.5%
Stingray	Termite mound smoke	24	11.8%
Stingray	Hot asphalt	1	0.5%
*Mandi*	Ice with salt	1	0.5%
*Piranha*	Copaíba (*Copaifera langsdorffii*) oil	1	0.5%
*Piranha*	Andiroba (*Carapa guianensis*) oil	1	0.5%
*Piranha*	Meracilina Phenoxymethyl penicillin (medicine) with copaíba oil	1	0.5%
Stingray	Warm water compress	1	0.5%
*Mandi*	Mercurochrome (medicine)	2	1%
*Mandi*	Meracilina with paracetamol (medicine)	1	0.5%
*Mandi*	Merthiolate with dipirona (medicine)	1	0.5%
**Total**		**44**	**22.5%**

Recovery time ranged from 2 weeks (51%) to 3 days (40.7%), or from 1-3 months (8.3%). There were sequelae concerning two *mandi*-related injuries, namely, a foot injury involving perforation, resulting in a loss of mobility of the hallux, and fragments of *mandi* spine left in the palmar region of the right hand.

Fishing requires physical strength in an industry dominated by males^3^ ^-^ ^6^, also observed in our study. Women are employed on a smaller scale in fishing. According to the interviewees, the fishermen’s wives worked mainly to repair fishing tackle. The interviewees’ level of schooling was found to be low, with an average of <9 years spent at school, which was similar to a report by Paraíba in 2013^7^. Most interviewees had started working as children, resulting in an early school dropout. 

All the fishermen lived in Cruzeiro do Sul, with 70% of the injuries having occurred in the Rio Juruá region that contains a great diversity of fish fauna. However, 30% of fish-related injuries occurred in the state of Amazonas, due to the displacement of these fishermen into neighboring areas.

The common names used to identify fish form part of local knowledge, allowing ready recognition of Amazonian fish species, and highlighting that each fish type has distinct natural history, strategies, and habits^8^. As well as identifying injuries caused by the mentioned species, it was possible to identify the fish at their lowest taxonomic level, which facilitated understanding of the injury dynamics.

A large number of injuries involved *mandis* (52.9%), *piranhas* (15.2%), and stingrays (9.8%), which is consistent with reports of a study involving artisanal fishermen from the middle Rio Araguaia region in Tocantins^6^. Injuries resulting from *mandis* were frequent, accounting for >50% of injuries, and all the interviewees reported having been injured on several occasions by this species. Stings on the tail of stingrays and the spines of catfish fins contain glandular tissue that produces toxins, which has been reported to be responsible for an intense inflammatory reaction^9^. *Piranhas* are known for their powerful bite and lacerating teeth that are used to capture their prey and act as a defense mechanism^1^.

The injured fishermen mostly sustained injuries to the upper limbs (63.7%), with a significant number of injuries to the hands (60.3%). This finding differs from other studies involving traumatic and venomous fish^3^ ^-^ ^5^, in which injuries more frequently involved the lower limbs. Pain and bleeding were reported in most cases, and can result in more serious injuries^5^ ^,^ ^10^.

Handling fish when using fishing gear lead to 97% of injuries. Direct contact with the fish increased the risk, as the interviewees stated they had not used protective equipment. In a study involving fishermen, conducted in Baía de Guanabara (Rio de Janeiro)^7^, only 31% used personal protective equipment, which was low, given fishing is a dangerous occupation posing several risks.

Most injuries occurred in the afternoon (46.6%) which was the most common reported period of work activity. Concerning seasonal injuries, 83.3% occurred in summer, a period that coincides with the receding waters of the Juruá River and a considerably increase in fishing activity.

A large number of reports within the National Disease Surveillance Data System refer to injuries sustained from aquatic fauna, of which a significant portion are attributed to fish^2^. In this study, 84% did not seek medical care, contributing to an under-reporting of cases in the Public Health System. Inadequate first aid and a lack of hospital care may lead to complications or sequelae^10^ ^-^ ^12^. In *mandi* injuries, sections of the *mandi* spine have been reported to break and penetrate a wound, requiring surgical removal^1^, and as was reported by one interviewee in our study. Concerning injuries involving catfish and stingrays, one study reported the need to remove spine fragments or stingers and to administer a tetanus vaccination and antibiotic therapy to reduce the risk of infection^13^.

One study involving fishermen from the Upper Paraguay River (Mato Grosso do Sul State)^11^ reported that fishermen still used many alternative herbal remedies, as well as harmful substances such as urine, which worsen the injury. In our study, the use of medicinal plants was reported, but termite smoke was the most prevalent remedy, and this form of treatment has been reported as widely employed in Rio Negro (Amazonas)^12^. Immersion in hot water was used by some interviewees to treat their injuries, and it has been shown to provide pain relief^14^. The use of *mandi* eyes as a remedy was also reported by injured fishermen in our study, similar to a report by Prado (2017)^14^ in a study undertaken at the Barra do Una Reserve (São Paulo). In that study, respondents reported having placed the eyes of the *mandi* fish over the wounded region to relieve the pain caused by the *mandi*’s spine. Some alternative remedies reported by the fishermen in our study have not been reported in the literature, and further studies are required to determine the efficacy of these alternative remedies.

Our results accorded with those of other studies, indicating the morbidity of fish injuries, and mainly involved venomous injuries. A lack of information regarding injury prevention and the circumstances surrounding the injuries was a primary finding. Consequently, injuries such as those reported in this study are likely to occur frequently. More detailed studies are required to determine the efficacy of certain alternative remedies; however, some alternative remedies undertaken were inadequate, as shown through limited first-aid understanding. Therefore, to reduce occupational risks, regulating working conditions for fishermen is required. Basic and secondary care are also essential to promote health and the treatment of injured fishermen who require advice concerning injury prevention, first-aid measures, and the importance of medical care.
